# Supplementary material for: A Novel Approach: Investigating the Intracellular Clearance Mechanism of Glyceraldehyde-Derived Advanced Glycation End-Products Using the Artificial Checkpoint Kinase 1 d270KD Mutant as a Substrate Model
Source: Cells. 2023 Dec 14;12(24):2838. doi: 10.3390/cells12242838 (PMC10741459; doi:10.3390/cells12242838)
Supplement: Supplementary file 1 [file cells-12-02838-s001.zip › cells-2745716-supplementary.pdf]

Supplementary Figure S1

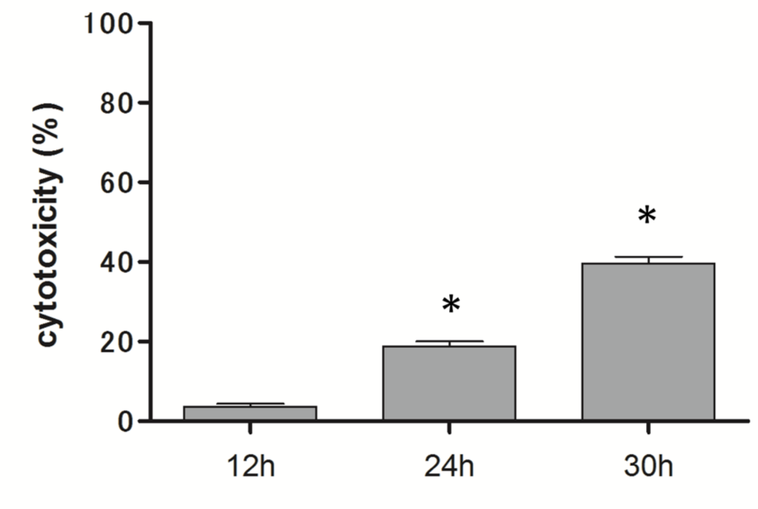

Figure S1. Lactate dehydrogenase (LDH) cytotoxicity assay. LDH levels in culture supernatants of HeLa cells treated with 2 mM GA. Data are representative of four independent experiments. Means ± SEM are shown. *$p < 0.001$ using one-way ANOVA with Dunnett's multiple comparisons.

Supplementary Figure S2

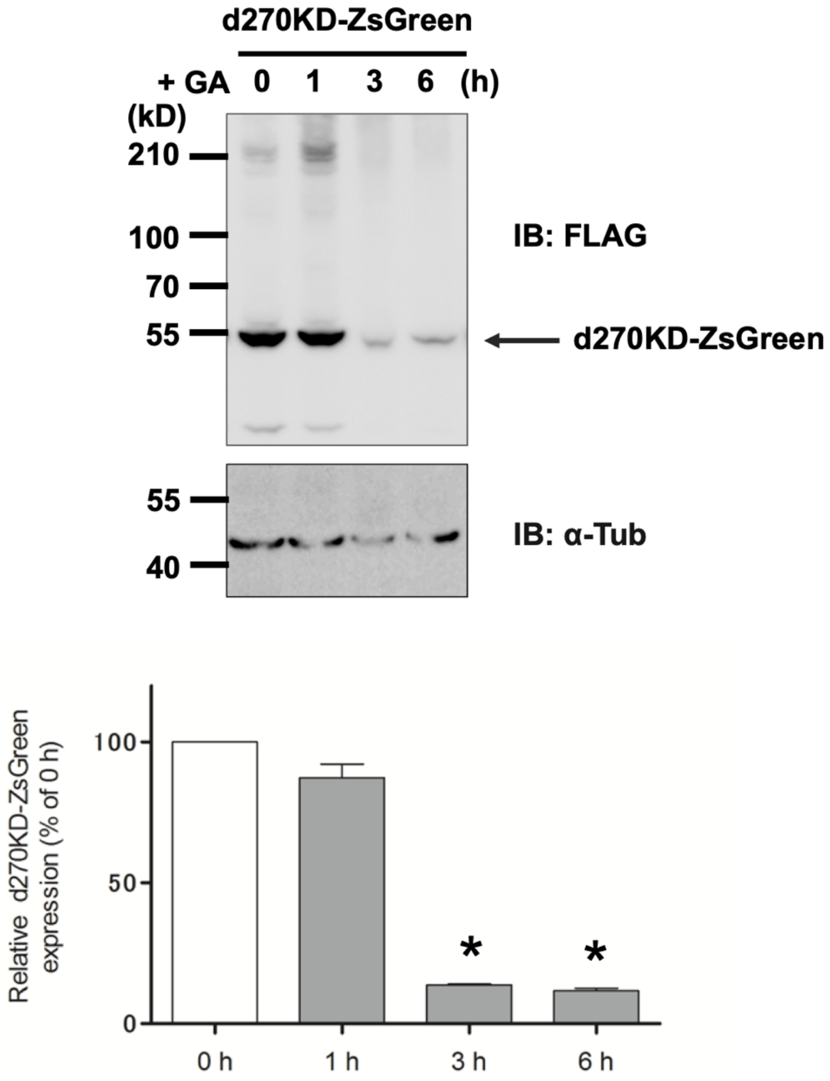

Figure S2. GA-stimulated rapid intracellular degradation of d270KD fusion proteins to ZsGreen1. The expression vector of d270KD fusion EGFP (d270KD-ZsGreen) was transfected into HeLa cells and stimulated with 2 mM GA 48 h later. Cell lysates were collected at the indicated times and subjected to a Western blot analysis. Densitometric quantification of d270KD-ZsGreen values relative to non-treatment group (0 h) are shown. Data were analyzed using one-way ANOVA followed by Dunnett's *post hoc* test and shown as mean values ± SEM of three independent experiments. *$p < 0.001$.

Supplementary Figure S3

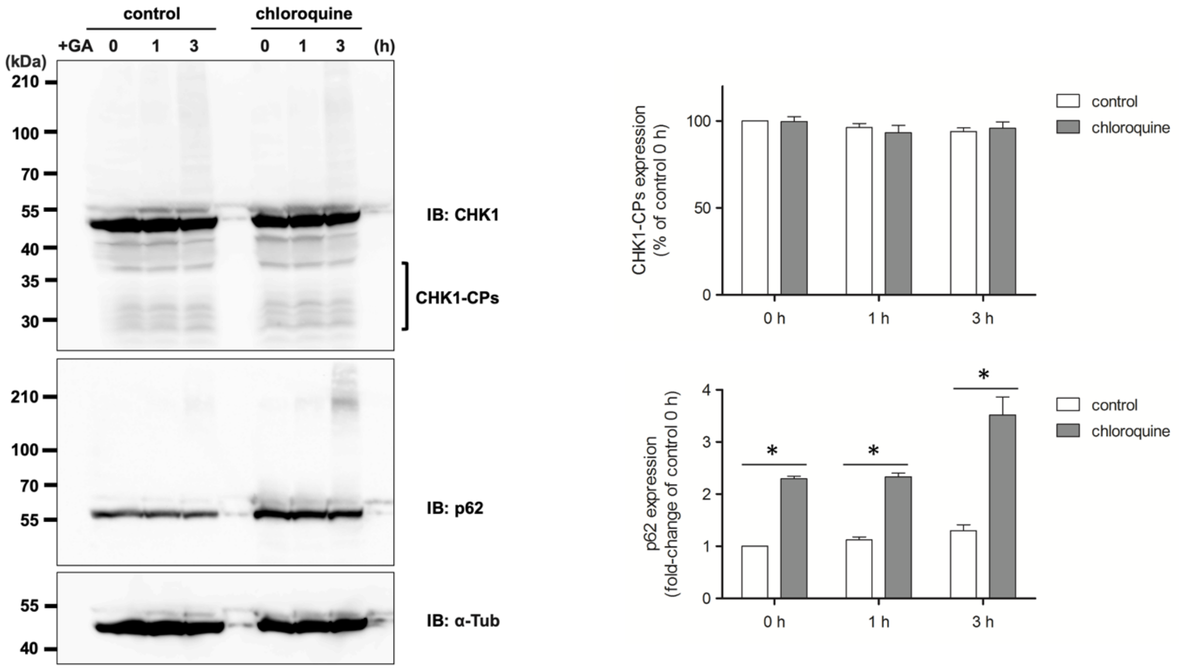

Figure S3. Effects of chloroquine on the GA-stimulated degradation of endogenous CHK1-CPs. HeLa cells were stimulated with 50 µM chloroquine for 20 h. Cells were collected after the stimulation with 2 mM GA for the indicated times. The resulting cell lysates were subjected to a Western blot analysis with the indicated antibodies. The densitometric quantification of CHK1 and total p62 values relative to the non-treatment control group (control 0 h) are shown. Data were analyzed using a two-way ANOVA followed by Bonferroni's *post hoc* test and shown as the mean values ± SEM of three independent experiments. *$p < 0.001$.
